# Two-Dimensional Copper/Nickel Metal–Organic Framework Nanosheets for Non-Enzymatic Electrochemical Glucose Detection

**DOI:** 10.3390/mi14101896

**Published:** 2023-09-30

**Authors:** Zhou Yao, Libing Zhang, Ting Wu, Haijun Song, Chengli Tang

**Affiliations:** 1 School of Mechanical Engineering, Zhejiang Sci-Tech University, Hangzhou 310018, China; yz7850@126.com; 2 College of Information Science and Engineering, Jiaxing University, Jiaxing 314001, China; songhaijun8837@126.com (H.S.); tcl-lily@mail.zjxu.edu.cn (C.T.); 3 Key Laboratory of Medical Electronics and Digital Health of Zhejiang Province, Jiaxing University, Jiaxing 314001, China

**Keywords:** metal–organic frameworks, copper/nickel nanosheets, non-enzymatic, electrochemical detection, glucose detection

## Abstract

Metal–organic frameworks (MOFs) have broad potential applications in electrochemical glucose detection. Herein, a green ultrasonic synthesis process is presented for preparing two-dimensional (2D) copper–nickel metal–organic framework nanosheets (CuNi-MOFNs) for glucose detection. The synthesized CuNi-MOFNs were characterized using scanning electron microscopy (SEM), scanning transmission electron microscope (STEM), X-ray diffractometer (XRD), and X-ray photoelectron spectrometer (XPS). The CuNi-MOFN nanocomposites were used to cover the glassy carbon electrode (GCE) and the CuNi-MOFNs-modified electrode was studied in alkaline media. Cyclic voltammetry (CV) and amperometric i–t curves indicated that the CuNi-MOFNs-modified electrode revealed great electrochemical performances towards glucose oxidation. Due to the ease of access to active metal sites in large specific surface of nanosheets, the CuNi-MOFNs-modified electrode can effectively improve the electronic transfer rate and enhance electrocatalytic activity of the CuNi-MOFNs-modified electrode. The CuNi-MOFNs-modified electrode showed electrochemical performances for glucose detection with a linear range from 0.01 mM to 4 mM, sensitivity of 702 μAmM^−1^cm^−2^, and detection limit of 3.33 μΜ (S/N = 3). The CuNi-MOFNs-modified electrode exhibited excellent anti-interference ability and high selectivity in glucose measurements. Hence, the CuNi-MOFNs-modified electrode has good, promising prospects in non-enzymatic electrochemical glucose detection.

## 1. Introduction

Diabetes has become one of the major chronic health diseases [1]. In addition, diabetes may induce other diseases, such as stroke, kidney failure, and cardiovascular disease. The amount of sugar intake is closely linked to diabetes, so sugar intake of daily diets should be controlled in daily life. Therefore, continuous and rapid detection of glucose concentration is of great significance for diabetes [2,3]. Due to its unique advantages of low cost, good selectivity, and anti-interference ability, electrochemical detection has become one of the most successful methods for quantitative detection of glucose. The two types of electrochemical glucose sensors are enzymatic glucose sensors and non-enzymatic glucose sensors [4,5]. Enzymatic glucose sensors are constrained by the high price of the necessary enzyme. In addition, temperature and pH are two major factors that could affect the activity of the enzyme and electrochemical behaviors during glucose detection [6]. Compared with enzymatic glucose sensors, non-enzymatic glucose sensors have the advantages of low cost, simple preparation process, high sensitivity, low detection limit, high stability, and good reproducibility. Therefore, non-enzymatic glucose sensors have attracted more attention by researchers [7,8].

Precious metals and alloys were widely used in non-enzymatic electrochemical glucose sensors [9,10]. Precious metals have inherent advantages such as good biocompatibility, good conductivity, and high sensitivity. However, they are still limited by the high cost of precious metals, which may greatly hinder their widespread application [11,12]. Metal oxides and hydroxides of copper or nickel have attracted more attention by researchers because of their excellent stability and electrochemical properties [13,14,15]. In addition to precious metals, metal oxides, and hydroxides in non-enzymatic glucose sensing, some transition metals, such as copper [16], cobalt [17], and nickel [18], have attracted more attention by researchers [19].

In recent years, researchers have increasingly focused on the bimetallic transition metal nanocomposites rather than single monometallic materials. Bimetallic transition metal nanocomposites have unique advantages such as low cost and high electrochemical activity in non-enzymatic glucose sensing. Bilal et al. [20] prepared an electrochemical glucose sensor based on polyaniline@CuNi nanocomposites to measure glucose oxidation. The prepared glucose sensor indicates great selectivity and a lower detection limit. Wang et al. [21] prepared an electrochemical glucose sensor based on Au@NiCo layered double hydroxide (LDH) core–shell nanostructures in a non-enzymatic glucose sensor. The fabricated electrochemical glucose sensor reveals great selectivity and great anti-interference ability.

Metal–organic frameworks (MOFs) are used in electrochemical sensors for glucose detection. MOFs have the unique advantages of various pores, a tunable structure, and great stability [22], MOFs are extensively used for many purposes, including catalysis [23], energy storage [24], and gas adsorption [25]. MOFs have great prospects in the application of electrochemical analysis [26]. However, despite these excellent characteristics, most bulk three-dimensional (3D) MOFs (such as ZIF-67 and MIL-101) usually have large grain sizes and poor conductivity. These shortcomings can limit mass transport of the material and reduce active sites interacting with MOFs [27,28]. Furthermore, it is crucial to obtain a large specific surface area of MOFs and maintain great catalytic performances by adopting a different synthesized method [29,30].

Two-dimensional (2D) metal–organic framework nanosheets (MOFNs) were characterized by large specific surface area, adjustable structure, and high surface-to-volume atom ratios [31]. Due to the excellent properties and features of 2D MOFNs, they could facilitate charge transfer and mass transport through the material [32,33]. Two-dimensional MOFNs are regarded as a promising kind of nanocomposites in non-enzymatic glucose detection [34]. Transition metals have great electrochemical activity and 2D MOFNs possess large specific surface area. Therefore, 2D MOFNs can be combined with different transition metals to construct new electrochemical glucose sensors [35]. Bimetallic MOFs effectively facilitate charge transfer efficiency and enhance electrochemical performances towards glucose oxidation. Therefore, 2D MOFN nanocomposites combined with the transition metals copper and nickel can further expose the active metal sites on 2D MOFNs and enhance catalytic efficiency in non-enzymatic glucose sensing [36].

In this work, copper–nickel metal–organic framework nanosheets (CuNi-MOFNs) were synthetized through a green ultrasonic synthesis method. Moreover, the effect of different ratios of copper and nickel on the electrochemical performances of the CuNi-MOFNs was investigated. The CuNi-MOFN nanocomposites were used to cover the glassy carbon electrode and studied in the alkaline media as modified material. The electrochemical behaviors of the CuNi-MOFNs-modified electrode in the quantitative detection of glucose were analyzed through Cyclic voltammetry (CV) and amperometric i–t curve. Meanwhile, the CuNi-MOFNs-modified electrode showed high selectivity and great reproducibility. In addition, the CuNi-MOFNs/GCE exhibited high sensitivity (702 μAmM^−1^cm^−2^), a wide linear range of 4 mM, and a lower detection limit (3.33 μΜ, S/N = 3). The CuNi-MOFNs-modified electrode exhibited promising prospects in non-enzymatic glucose detection.

## 2. Experimental Section

### 2.1. Materials

Copper chloride dihydrate (CuCl_2_·6H_2_O), ethanol, fructose (Fru), N, N-dimethylformamide (DMF), sodium hydroxide (NaOH), and glucose (Glu) were purchased from Sinopharm Chemical Reagent Co., Ltd. (Shanghai, China). Nickel chloride hexahydrate (NiCl_2_·6H_2_O), p-phthalic acid (PTA), uric acid (UA), ascorbic acid (AA), sodium chloride (NaCl), and triethylamine (TEA) were obtained from Aladdin Biochemical Technology Co., Ltd. (Shanghai, China). All chemicals were of analytical grade and used without further treatment, and deionized water was used throughout the experiments.

### 2.2. Synthesis Process of CuNi-MOFNs

**Figure 1:**
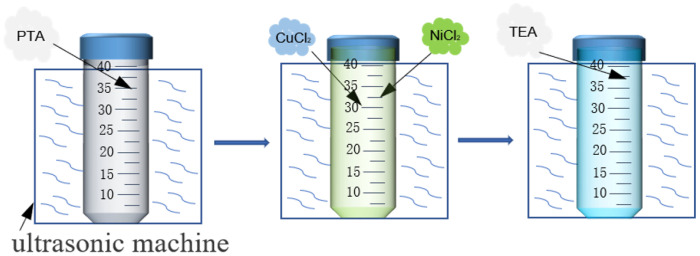
The preparation process of CuNi-MOFNs.

The CuNi-MOFNs were prepared as shown in Figure 1. First, 25 mL DMF were poured into the 40 mL centrifuge tube. An amount of 0.062 g PTA was added into the centrifuge tube and ultrasonic treatment was performed for 20 min to obtain a transparent solution. Next, 0.48 mmol CuCl_2_ and 0.16 mmol NiCl_2_ (Cu/Ni 3:1) were added into the mixed solution and sonicated for 30 min. When the above solution became clear, 400 μL TEA was added into the centrifuge tube. The mixed solution was quickly transferred into the ultrasonic machine, and the centrifuge tube was shaken under water level. The above solution was sonicated for 10.5 h under air conditions. The mixture products were centrifuged four times. Finally, the blue-green composites were washed with ethanol and dried at 80 °C overnight to obtain the CuNi-MOFNs. Furthermore, the CuNi-MOFNs were prepared with different ratios (Cu/Ni 2:1 and 4:1) using the same method without adding NiCl_2_·6H_2_O.

### 2.3. Fabrication of CuNi-MOFNs/GCE

Before the modification of the glassy carbon electrode (GCE), the GCE (diameter d = 0.3 cm) was polished with 300 nm and 50 nm alumina slurry, respectively. The GCE was sonicated with ethanol and deionized water, separately. Then, it was dried at room temperature. An amount of 3 mg of the as-synthetized material was dissolved in 10 μL Nafion solution (0.05 wt%) and 1 mL deionized water. The mixed solution underwent ultrasonic treatment for 30 min to obtain a uniform solution. Finally, a drop of suspension solution was casted on the electrode surface and dried overnight to obtain the CuNi-MOFNs-modified electrode.

### 2.4. Characterization

The microstructure of the synthesized CuNi-MOFN nanocomposites was characterized by scanning electron microscopy (SEM, MAGELLAN-400, FEI Company, Hillsboro, OR, USA). Elemental compositions of the as-prepared samples were investigated using scanning transmission electron microscope (STEM, FEI Talos F200X, FEI Company, Hillsboro, OR, USA). The crystal phases structures of the Cu-MOFNs, Ni-MOFNs, and the CuNi-MOFNs were analyzed by an X-ray diffractometer (XRD, DX-2700BH, Dandong Haoyuan Instrument, Dandong, China) with 2θ value between 5° to 65°. X-ray photoelectron spectrometer (XPS, ESCALAB 250Xi, Thermo Fisher Scientific, Waltham, MA, USA) analysis investigated the elemental compositions of the CuNi-MOFN nanocomposites. The surface area of the synthesized CuNi-MOFNs was revealed by specific surface area analyzer (NOVAtouch LX-4, Quantachrome Instruments, Boynton Beach, FL, USA). Electrochemical tests were carried out using an electrochemical workstation (RST5000, Suzhou, China) with 0.1 M NaOH solution. Working electrode, counter electrode and reference electrode were used as CuNi-MOFNs-modified electrode, platinum wire, and mercury oxide (HgO), respectively, in the typical three-electrode system.

## 3. Results and Discussion

### 3.1. Morphology and Structures of CuNi-MOFNs

**Figure 2:**
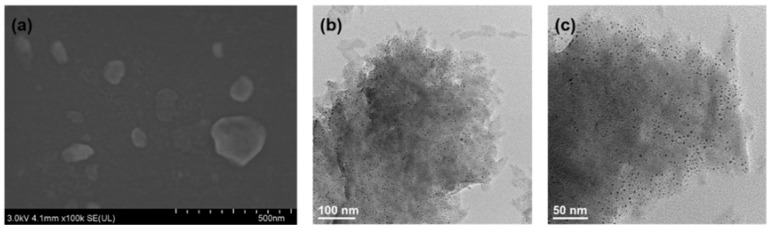
(**a**) SEM image of the as-synthesized CuNi-MOFNs. (**b**,**c**) TEM images of the as-synthesized CuNi-MOFNs.

**Figure 3:**
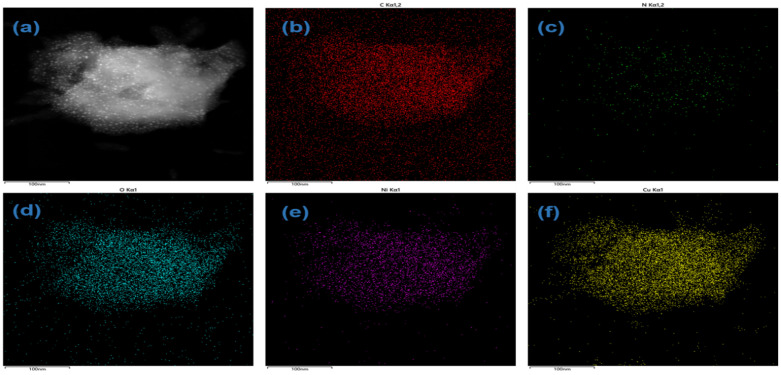
(**a**) STEM image of the as-synthesized CuNi-MOFNs. (**b**–**f**) Elemental mapping image of C, N, O, Ni, and Cu among the as-synthesized CuNi-MOFNs.

**Figure 4:**
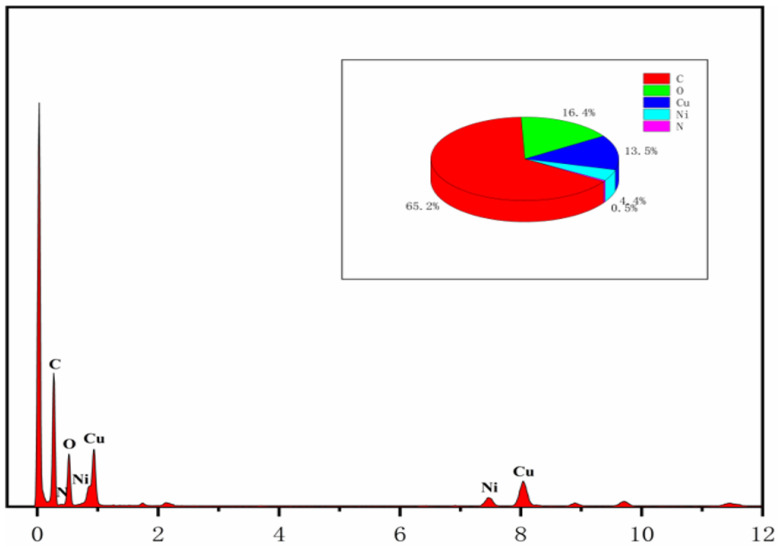
EDX spectrum of the as-synthesized CuNi-MOFNs.

**Figure 5:**
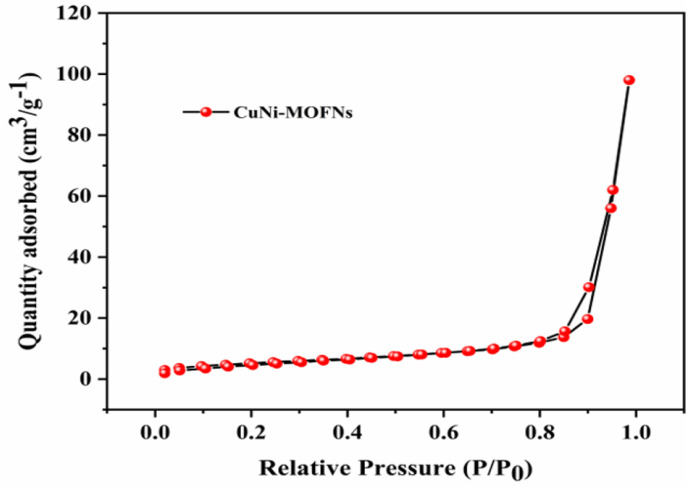
N2 isothermal adsorption and deposition curve of the as-synthesized CuNi-MOFNs.

Surface morphology of the CuNi-MOFN nanocomposites was analyzed by SEM. Through SEM image observation, the CuNi-MOFNs exhibited the 2D nanosheet microscopic morphology shown in Figure 2a. The microstructure of the CuNi-MOFNs was revealed using TEM. TEM images further demonstrated that the CuNi-MOFNs possess the structure of 2D nanosheets, as shown in Figure 2b,c. It revealed that the structures of CuNi-MOFNs have rich pores/voids, as shown in Figure 3a. By analyzing samples in Figure 3b–f, it was found that the CuNi-MOFN nanocomposites contain five elements of C, N, O, Ni, and Cu. The EDX image was acquired in STEM mode, as shown in Figure 4. The results show the distribution and proportions of five elements in the as-synthetized CuNi-MOFNs. The CuNi-MOFN nanocomposites were measured by the N2 isothermal adsorption and deposition curve at 77.35 K, as shown in Figure 5. BET surface area of CuNi-MOFNs was 34.7 m^2^/g. A relatively large surface area can expose active metal sites in the CuNi-MOFNs and promote surface interactions, which can play a significant role in non-enzymatic electrochemical glucose detection.

**Figure 6:**
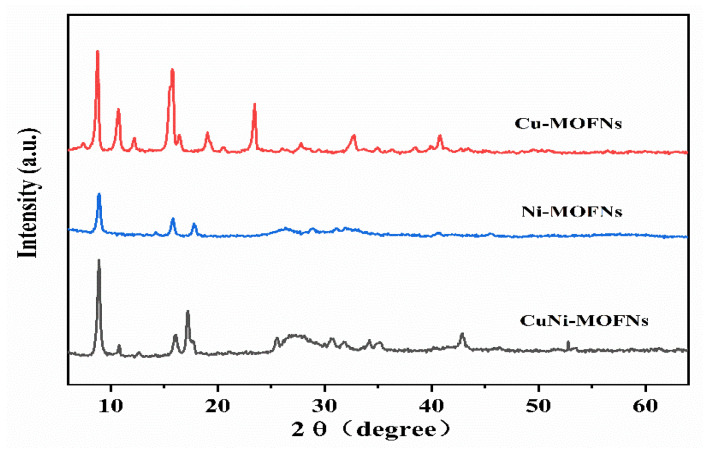
XRD patterns of the Cu-MOFNs, the Ni-MOFNs, and the CuNi-MOFNs, respectively.

The crystal structure of the CuNi-MOFN nanocomposites was investigated by XRD. The Cu-MOFNs, the Ni-MOFNs, and the CuNi MOFNs sample images are displayed in Figure 6. The image of the Ni-MOFNs exhibits two diffraction peaks at 9.5° and 16.3°, respectively. These two peaks are associated with the (100) and (101) planes [37]. Compared with the Cu-MOFNs, the diffraction peaks of the CuNi-MOFNs have weakened at 11.6°, 13.1°, and 24.3°. This may be because Ni ions replaced Cu ions and bounded with p-phthalic acid.

**Figure 7:**
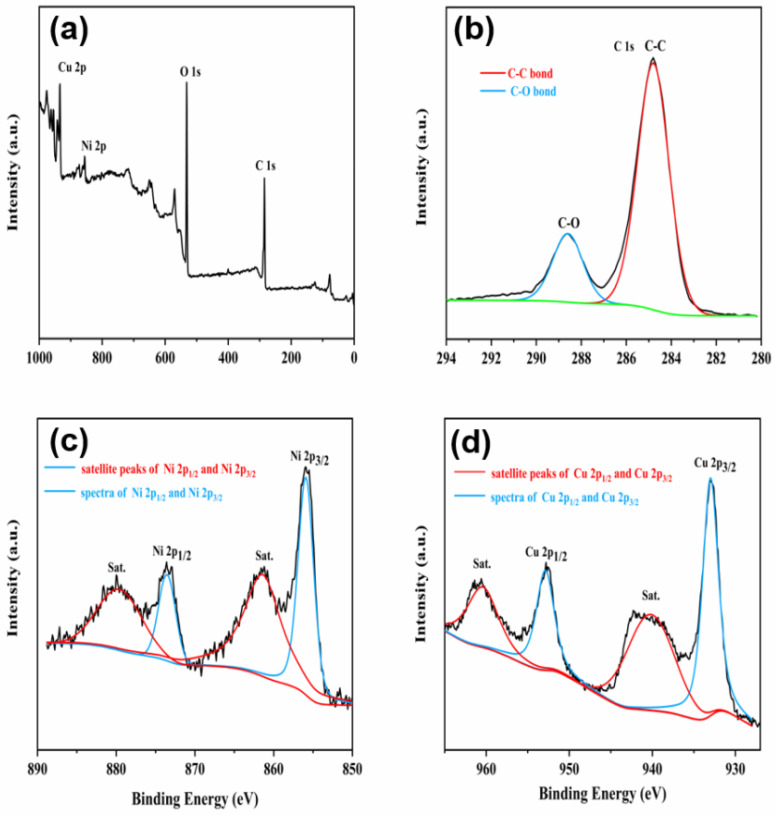
(**a**) Full XPS spectrum. (**b**) Core-level spectra of C 1s, (**c**) core-level spectra of Ni 2p, and (**d**) core-level spectra of Cu 2p of the as-synthesized CuNi-MOFNs.

Element compositions and electronic states of the CuNi-MOFNs were analyzed using XPS. From the survey spectrum in Figure 7a, the full XPS spectrum demonstrated that the as-prepared CuNi-MOFNs indicated the existence of Cu, Ni, C, and O elements. In Figure 7b, the C 1s spectrum revealed two evident peaks centered at 284.8 eV and 288.6 eV, corresponding to the C–C and C–O bonds, respectively [38]. In Figure 7c, the core-level spectra of Ni 2p indicated two distinct peaks at 855.9 eV and 873.5 eV, corresponding to the binding energies of the Ni 2p_3/2_ and Ni 2p_1/2_ spectra, respectively. In addition, there is a pair of related satellite peaks at 861.4 eV and 879.6 eV, which correspond to Ni 2p_3/2_ and Ni 2p_1/2_. This further demonstrated that nickel hydroxide contains Ni^2+^ [39]. In Figure 7d, the core-level spectrum of Cu 2p presented two main peaks for the binding energies of Cu 2p spectrum, located at 933.2 eV and 952.9 eV, respectively, which correspond to Cu 2p_3/2_ and Cu 2p_1/2_. Furthermore, the binding energies of the Cu 2p spectrum were centered at 940.2 eV and 960.4 eV, which can be ascribed to the related satellite peaks of the Cu 2p spectrum [40]. Therefore, the experimental results indicate the existence of Cu^2+^ in the as-synthesized CuNi-MOFNs.

### 3.2. Electrochemical Characterizations of CuNi-MOFNs

**Figure 8:**
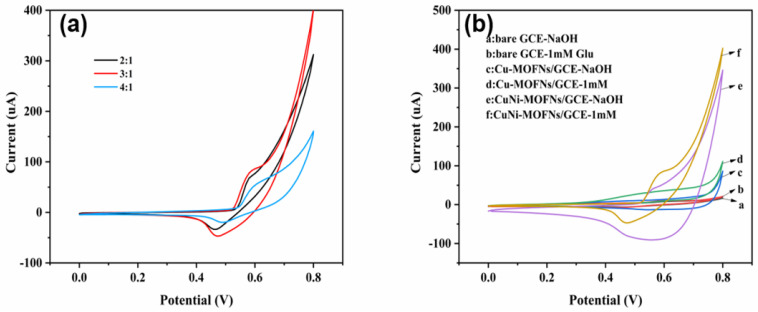
(**a**). Cyclic voltammetry curves of the CuNi-MOFNs/GCE containing 1 mM glucose at the same scan rate (50 mV/s) in 0.1 M NaOH solution (**b**). CVs of the bare GCE, the Cu-MOFNs/GCE, and the CuNi-MOFNs/GCE in the absence and presence of 1.0 mM glucose in 0.1 M NaOH solution with the same scan rate (50 mV/s).

**Figure 9:**
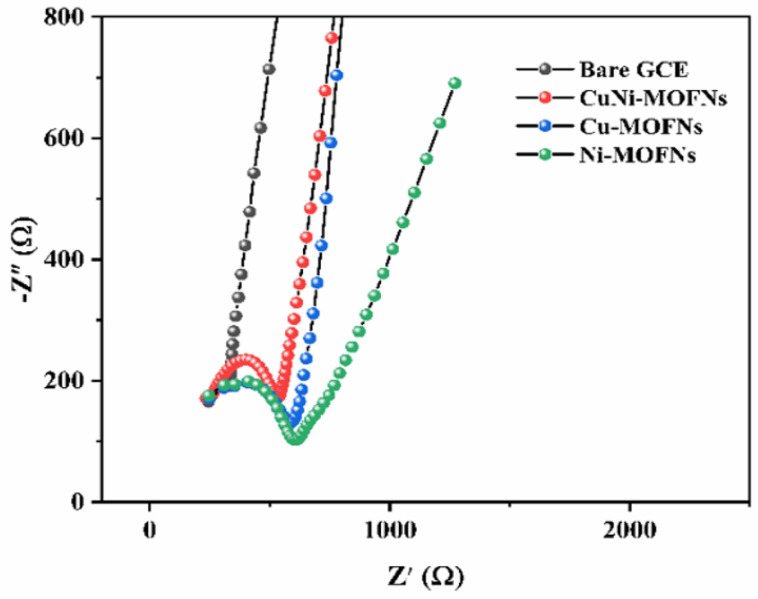
Impedance spectra of the bare GCE, the Cu-MOFNs/GCE, the Ni-MOFNs/GCE and the CuNi-MOFNs/GCE in 5 mM K_3_[Fe (CN)_6_] and 0.1 M KCl.

The CV studies were performed in the potential window of 0~0.8 V at the same scan rate (50 mV/s) in 0.1 M NaOH solution. The effects of different ratios of copper and nickel on the electrochemical behaviors of the as-synthesized CuNi-MOFNs were analyzed. The electrocatalytic performances of the CuNi-MOFNs-modified electrode towards glucose oxidation were examined in 0.1 M NaOH solution. The ratio of Cu/Ni for the as-synthesized CuNi-MOFNs/GCE was 3:1. The CuNi-MOFNs-modified electrode revealed a distinct oxidation peak, as shown in Figure 8b. A distinct pair of peak currents were observed at 0.58 V and 0.43 V in the 0.1 M NaOH solution in the absence and presence of 1.0 mM glucose, respectively, with the same scan rate (50 mV/s). Therefore, the CuNi-MOFN nanocomposites were used for the subsequent experimental test. As indicated in Figure 8b, the CV curves of the bare GCE are shown in the absence and presence of 1.0 mM Glu in bending line a and b, respectively, while the CV curves of the Cu-MOFNs/GCE and the CuNi-MOFNs/GCE are shown in bending line c and d and bending line e and f, respectively. The oxidation peak current of the bare GCE had a very weak peak, which indicated less glucose oxidation. Obviously, the oxidation peak current of CuNi-MOFNs/GCE was much higher than Cu-MOFNs/GCE in the 1 mM glucose. This result demonstrates that CuNi-MOFNs/GCE can effectively promote charge transfer and further enhance electrochemical activity. Cu(OH)_2_ and Ni(OH)_2_ can convert CuOOH and NiOOH by reacting with OH, respectively. CuOOH and NiOOH can then oxidize glucose into gluconolactone [41]. Nyquist plots were used to evaluate the conductivity of the modified electrode. Nyquist plots of the bare GCE, the Cu-MOFNs/GCE, the Ni-MOFNs/GCE and the CuNi-MOFNs/GCE in 5 mM K_3_[Fe (CN)_6_] and 0.1 M KCl from 0.01 Hz–100 kHz are shown in Figure 9. The charge transfer resistance of the CuNi-MOFNs-modified electrode was lower than the Cu-MOFNs/GCE and the Ni-MOFNs/GCE. Therefore, the CuNi-MOFNs/GCE can effectively facilitate charge transfer and improve conductivity.

**Figure 10:**
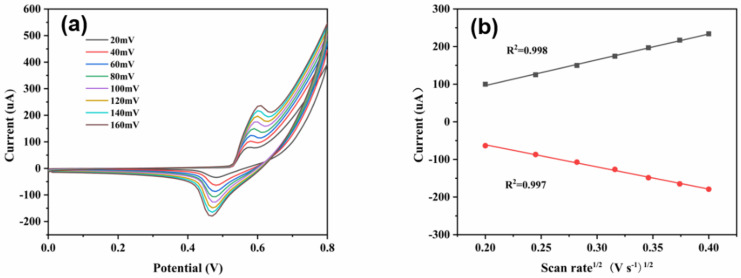
(**a**) The electrochemical performances of the CuNi-MOFNs/GCE at various scan rates (20 mV/s~160 mV/s) in 0.1 M NaOH solution. (**b**) The corresponding linear relationship of the CuNi-MOFNs/GCE between peak current and square root of scan rates.

The CuNi-MOFNs-modified electrode demonstrated significant electrocatalytic activity for glucose sensing. The copper content may be hydrolyzed, and the peak potentials lightly moved towards the positive and negative directions with the increasing scan speeds [42]. The electrochemical performances of the CuNi-MOFNs/GCE with various scan rates (from 20 mV/s to 160 mV/s) were analyzed in 0.1 M NaOH solution including 1.0 mM glucose. As the scan rates increased, the anodic peak currents and reduction peak currents also increased, as shown in Figure 10a. The results indicate that the peak currents of the oxidation and reduction were closely related to the square root of the scan rates, as shown in Figure 10b. This indicated that the representative diffusion-controlled electrochemical process is a perfect method for glucose oxidation [20].

**Figure 11:**
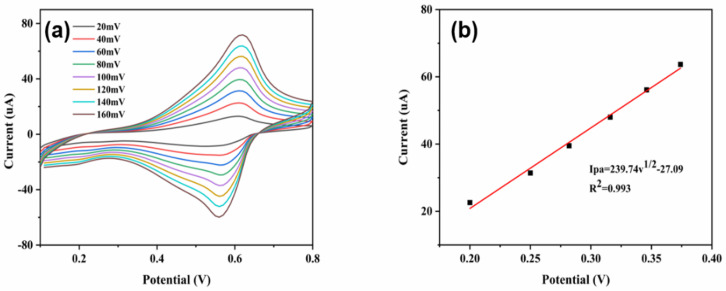
(**a**) CV curves of the ECSA of the CuNi-MOFNs/GCE in 5 mM K3[Fe (CN)6] electrolyte containing 0.1 M KCl at various scan rates (from 20mV/s to 160mV/s). (**b**) Corresponding linear relationship between anodic peak currents (*Ipa*) and square root of scan rates (*v*^1/2^).

The Randles-Sevcik equation was used to calculate the electrochemical surface area (ECSA) of the as-prepared CuNi-MOFNs-modified electrode [43]. The ECSA of the CuNi-MOFNs-modified electrode was performed with different scan rates, from 20 mV/s to 160 mV/s, in 5 mM K_3_[Fe (CN)_6_] containing 0.1 M KCl through the cyclic voltammetry method, as shown in Figure 11a. The CV performances of the CuNi-MOFNs-modified electrode and the fitting curve of the ECSA are shown in Figure 11b. According to Equation (1), the ECSA of the CuNi-MOFNs/GCE is approximately 0.05 cm^2^.

(1) $$I_{pa}=269,000\times A\times n^{3/2}\times D_{0}{}^{1/2}\times C_{0}\times v^{1/2}$$

In the above equation, *I_pa_* stands for anodic peak current, *A* stands for ECSA (cm^2^), *n* stands for electron transfer number (*n* = 1), *D*_0_ stands for diffusion coefficient of K_3_ [Fe (CN)_6_], *C*_0_ stands for the redox probe concentration in bulk solution, and *v* stands for scan rate (V s^−1^)

**Figure 12:**
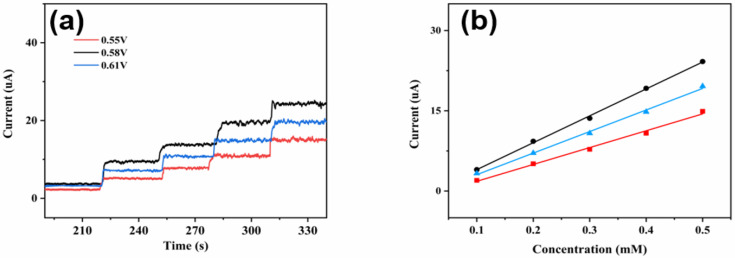
(**a**) Current responses of the CuNi-MOFNs/GCE at various potentials with continuous addition of 0.1 mM glucose in 0.1 M NaOH solution. (**b**) The linear relationships of the fitting curves at various potentials.

The effect of the CuNi-MOFNs-modified electrode towards glucose oxidation was investigated. A suitable potential can accelerate the charge transfer and improve catalytic efficiency. The effects of various potentials on current responses at 0.55 V, 0.58 V, and 0.61 V were studied, respectively. Figure 12a indicates that the current responses of the CuNi-MOFNs-modified electrode were carried out with continuous addition 0.1 mM of glucose in 0.1 M NaOH solution under continuous stirring. It reveals that current responses also increased with the increasing of glucose concentrations. By analyzing Figure 12b, the fitting curve between current responses and glucose concentrations can be seen. According to experimental analysis, the maximum slope of current response was generated at 0.58 V. At this potential, the CuNi-MOFNs-modified electrode indicated great selectivity on current response. Therefore, the optimized potential was selected as 0.58 V, which was used for the subsequent study of electrochemical glucose detection.

**Figure 13:**
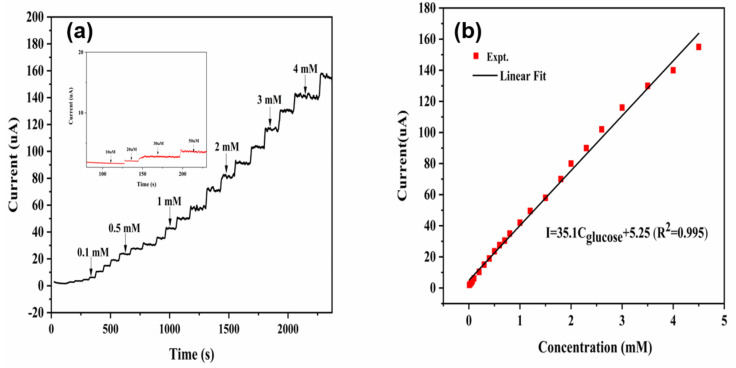
(**a**) Current responses of the CuNi-MOFNs-modified electrode upon successive additions of glucose concentrations. (**b**) The calibration curve of the CuNi-MOFNs-modified electrode between current responses and glucose concentrations with optimized potential of 0.58 V in 0.1 M NaOH solution.

The electrochemical behavior of the CuNi-MOFNs-modified electrode was investigated. All i–t curves on current responses were carried out in 0.1 M NaOH solution under continuous stirring with the optimized potential of 0.58 V. The current responses of the CuNi-MOFNs-modified electrode to the continuous additions of glucose with various concentrations were examined in 0.1 M NaOH solution. The CuNi-MOFNs/GCE exhibited a sensitive increase with continuous additions of glucose, as shown in Figure 13a. Current responses were proportional to the glucose concentration. Furthermore, the inset plot in Figure 13a reveals successive additions of glucose on current responses at lower concentrations. The experimental results indicate that the CuNi-MOFNs-modified electrode exhibited great electrochemical activity towards glucose oxidation. The CuNi-MOFNs/GCE was tested in the range from 0.01 mM to 4 mM with successive additions of glucose concentration, as shown in Figure 13b. The corresponding calibration curve is I = 35.1 C_glucose_ (mM) + 5.25 (R^2^ = 0.995), where C_glucose_ represents the glucose concentration. It reveals a great linear relation between the current responses and glucose concentrations of the CuNi-MOFNs-modified electrode. The sensitivity was calculated according to Equation (2) [22]. The sensitivity of the CuNi-MOFNs/GCE is 702 μAmM^−1^cm^−2^ and the lower detection limit (LOD) was calculated on the basis of a three times signal-to-noise ratio (S/N = 3). According to the above calibration curve, the LOD of the CuNi-MOFNs-modified electrode was 3.33 μM.

(2) $$S=\frac{m}{A}$$

In the above equation, *S* represents the sensitivity of the CuNi-MOFNs/GCE, *m* represents the slope of the calibration curve of the CuNi-MOFNs/GCE, and *A* represents the electrochemical active area of the CuNi-MOFNs/GCE.

**Figure 14:**
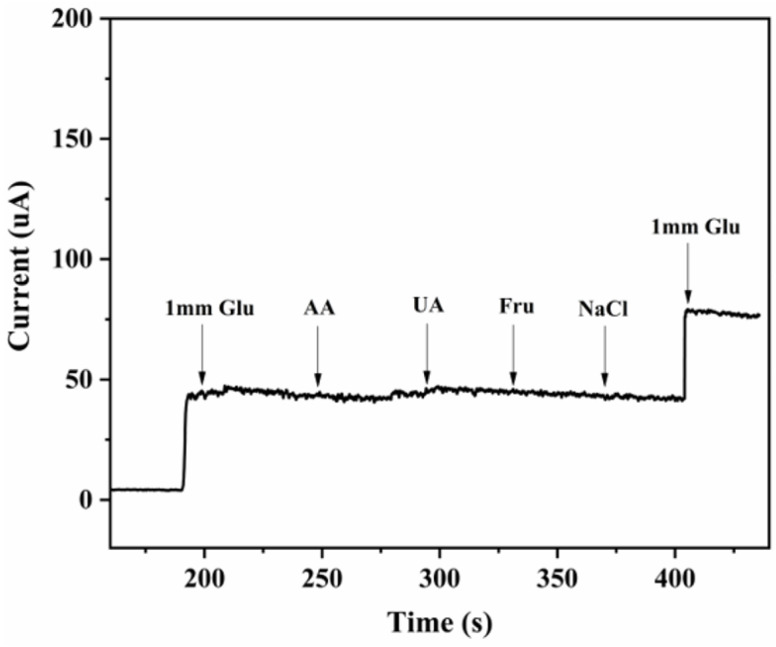
Amperometric responses of the as-prepared CuNi-MOFNs/GCE with the addition of 1 mM glucose and various interfering species (0.1 mM ascorbic acid, 0.1 mM uric acid, 0.1 mM fructose, and 0.1 mM sodium chloride).

The as-prepared CuNi-MOFNs/GCE was further examined for selectivity and anti-interference ability to evaluate glucose sensing. Anti-interference ability is the ability to distinguish glucose from interfering species, which is commonly important in practical detection of any biosensors. Other interfering species, such as AA, UA, Fru, and NaCl, were selected to prove selectivity and anti-interference ability of the current responses of the CuNi-MOFNs/GCE. Figure 14 exhibits the amperometric responses of the CuNi-MOFNs/GCE to continuous additions of glucose (1 mM), 0.1 mM ascorbic acid, 0.1 mM uric acid, 0.1 mM fructose, and 0.1 mM sodium chloride in 0.1 M NaOH solution under continuous stirring. The current response for adding 1 mM glucose was noticeable, and the current increment was much smaller for the interfering species. These results demonstrate that the CuNi-MOFNs-modified electrode has great anti-interference ability and high selectivity in glucose sensing.

The electrochemical performance of the CuNi-MOFNs-modified electrode compared with other reported and presented electrochemical non-enzymatic glucose sensors is shown in Table 1. The result exhibited that the CuNi-MOFNs-modified electrode revealed a wider linear range than other non-enzymatic glucose sensors towards glucose oxidation. The reason for this result was Ni/Cu active metal sites in a large specific surface area of nanosheets in the prepared CuNi-MOFNs/GCE. Therefore, 2D copper–nickel metal–organic framework nanosheets have good potential for quantitative detection of glucose as modifications of the electrode.

## 4. Conclusions

In summary, copper–nickel metal–organic framework nanosheets were synthesized as modified electrode in a non-enzymatic glucose sensor through a rapid and simple ultrasonic synthesis method. The synthesized copper–nickel metal–organic framework was successfully applied for the sensitive determination of the glucose detection under alkaline media. The cyclic voltammetry and amperometric i–t curves of the CuNi-MOFNs/GCE reveal great electrochemical activity towards glucose oxidation. Due to the ease of access to active metal sites in the large specific surface of the nanosheets, a CuNi-MOFNs-modified electrode could promote the electronic transfer and improve the electrocatalytic reaction. The optimized potential was selected as 0.58 V. The results indicate that the as-prepared CuNi-MOFNs/GCE had high selectivity and anti-interference ability in glucose measurement. The CuNi-MOFNs-modified electrode demonstrated great electrochemical performance in glucose sensing in the range from 0.01 mM to 4 mM, the sensitivity of 702 μA mM^−1^cm^−2^, and the LOD of 3.33 μΜ (S/N = 3). Moreover, the CuNi-MOFNs/GCE modified electrode had great anti-interference ability in glucose sensing. Therefore, 2D CuNi-MOFNs-modified electrodes are promising candidates for non-enzymatic electrochemical glucose detection.

## Figures and Tables

**Table 1:** Comparison of the CuNi-MOFNs-modified electrode with other reported and presented non-enzymatic glucose sensors.

Electrode	Sensitivity (μA mM^−1^ cm^−2^)	Linear Range (mM)	Detection Limit (μM)	References
CuNi-MOFNs	702	0.01–4	3.33	This work
CuO/NiO-C/CT	586.7	0.0001–4.5	0.037	[43]
Ni-Cu/TiO_2_ NTs	1590.9	0.01–3.2	5	[44]
Ni-Co PBA HNCs	149	0.002–3.79	1.2	[45]
Co-Ni nanorods	544	0.1–1	~	[46]
NiO/Co_3_O_4_@C	690	0.005–4	2.28	[47]
Co/Zn-MOF	833.6	up to 5	6.5	[48]

## Data Availability

Not applicable.
